# Tin, Titanium, Tantalum, Vanadium and Niobium Oxide Based Sensors to Detect Colorectal Cancer Exhalations in Blood Samples

**DOI:** 10.3390/molecules26020466

**Published:** 2021-01-17

**Authors:** Michele Astolfi, Giorgio Rispoli, Gabriele Anania, Elena Artioli, Veronica Nevoso, Giulia Zonta, Cesare Malagù

**Affiliations:** 1 Department of Physics and Earth Sciences, University of Ferrara, 44122 Ferrara, Italy; stlmhl@unife.it (M.A.); zntgli@unife.it (G.Z.); 2 SCENT S.r.l (SME company), Via Quadrifoglio 11, 44124 Ferrara, Italy; 3 Department of Neuroscience and Rehabilitation, University of Ferrara, 44121 Ferrara, Italy; rsg@unife.it; 4 Department of Medical Sciences, University of Ferrara, 44121 Ferrara, Italy; ang@unife.it (G.A.); elena01.artioli@student.unife.it (E.A.); veronica.nevoso@student.unife.it (V.N.)

**Keywords:** nanostructured sensors, tumor markers, colorectal cancer, blood, chemoresistivity, volatile organic compounds

## Abstract

User-friendly, low-cost equipment for preventive screening of severe or deadly pathologies are one of the most sought devices by the National Health Services, as they allow early disease detection and treatment, often avoiding its degeneration. In recent years more and more research groups are developing devices aimed at these goals employing gas sensors. Here, nanostructured chemoresistive metal oxide (MOX) sensors were employed in a patented prototype aimed to detect volatile organic compounds (VOCs), exhaled by blood samples collected from patients affected by colorectal cancer and from healthy subjects as a control. Four sensors, carefully selected after many years of laboratory tests on biological samples (cultured cells, human stools, human biopsies, etc.), were based here on various percentages of tin, tungsten, titanium, niobium, tantalum and vanadium oxides. Sensor voltage responses were statistically analyzed also with the receiver operating characteristic (ROC) curves, that allowed the identification of the cut-off discriminating between healthy and tumor affected subjects for each sensor, leading to an estimate of sensitivity and specificity parameters. ROC analysis demonstrated that sensors employing tin and titanium oxides decorated with gold nanoparticles gave sensitivities up to 80% yet with a specificity of 70%.

## 1. Introduction

Nowadays, cancer is a ubiquitous pathology of crucial impact, and it is expected to rank as the leading cause of death worldwide in the 21st century. Its incidence and mortality are progressively increasing due to the fast population growth and the increase in average age. For this reason, early cancer detection is a challenge for many researchers working in many different fields, and different techniques have been implemented to allay the cancer threat. The main techniques proposed till now range from the main risk factor reduction to longer standing screening and early detection programs [1]. The reduced mortality for some types of cancer, following the adoption of these practices [2], is forcing the scientific community to develop more and more efficient screening techniques and follow-up protocols, in particular to defeat one of the most common localized cancer pathologies, such as colorectal cancer (CRC).

CRC has an important impact on public health, being the third most common malignancy for incidence and second for mortality worldwide [3] accounting for 11% of all cancer diagnoses. It affects people of both sexes, typically aged 65 or over [4] and its symptoms are various, although it may be asymptomatic for a long time, so delaying its detection. The most common CRC symptoms are: rectal bleeding, followed by chronic blood loss leading to physical fatigue and anemia [5]. Usually, endoscopic procedures are recommended as first investigation if CRC is suspected, allowing to collect specimens from presumable tumor lesions and to obtain a reliable and suitable anatomopathological examination (cancer diagnosis validation and histological characterization) [6].

The nowadays available CRC screening techniques, crucial in early-stage tumor detection, are divided into non-invasive and invasive tests. The main non-invasive tests are:

- Guaiac-based fecal occult blood test (gFOBT) and the newer fecal immunochemical test (FIT), based on the detecting blood in stools through chemical and immunochemical reactions respectively [7,8];
- The novel multitarget stool DNA (MT-sDNA), combining FIT and altered DNA biomarkers detection in CRC cells entrapped into the stool [7,8];
- Radiologic examinations including capsule endoscopy [9] and Computed tomographic colonography (CTC) [10].

The most widely used invasive tests are the flexible sigmoidoscopy (FS) and colonoscopy that, in addition to the direct visualization, allow to collect a pathology specimen. For instance, Italian guidelines recommend pancolonoscopy or alternatively recto-sigmoidoscopy associated with virtual colonoscopy and double contrast barium enema [11,12]. European Member States recommend to men and women with ages ranging between 50 and 74 years to undergo screening programs, such as annual or biennial FOBTs, eventually followed by colonoscopy in case of positive results [13]. FIT is the most used screening test worldwide, because it is particularly user friendly, more reliable than gFOBT and does not require dietary restrictions, though it counts a 65% of false positives (due to non-tumoral bleedings) so leading to a huge amount of non-operative colonoscopies [14]. 

In the last years, many efforts have been made by the research community in CRC screening, introducing new solutions that improve the efficiency and reliability of CRC detection in respect to the commonly used aforementioned techniques. The most common approach is to detect CRC by analyzing easily collectable biological samples (by means of low invasive processes) such as urine, breath, blood, serum and stools. In the last decade, the technologies developed for this scope have shown a significant clinical impact, improving the understanding of disease genesis, the related processes and its evolution [15].

Many current studies demonstrate that neoplastic lesions, such as CRC, emit specific VOCs (volatile organic compounds, such as benzene, alkanes, aldehydes or their derivatives), that can be exploited as reliable cancer markers [16]. VOCs are generally wastes discharged in a living organism, produced by the altered metabolism of cancer cells and, their detection is nowadays a promising and popular topic among researchers. With this aim, the company SCENT S.r.l. started a project in 2014 to detect VOCs exhaled by stool samples by using a hand-made prototype, named SCENT A1, based on an array of five MOX sensors, to identify CRC presence [14,17,18,19,20]. The reproducibility, the reliability, the non-invasiveness, and the quickness in detecting CRC exhibited by SCENT A1, made it suitable to be combined to the current screening methods, sensibly improving their ability to detect this pathology.

A second hand-made prototype, named SCENT B1 [21], was developed from the same company, to detect VOCs in CRC human tissue samples, cell cultures [22] and blood [23]. Encouraging results were achieved in the human tissue application where the statistical analysis of its sensor responses, performed by using principal component analysis approach, showed the SCENT B1 ability to discriminate between healthy and tumor samples [24]. Moreover, this device was even able to identify the tumor grade, that assesses the undifferentiation level of the tumor cells [24]. On this promising basis, knowing that neoplasms are highly vascularized lesions discharging a substantial amount of VOCs in the blood stream [25], the SCENT B1 device is used here to detect VOCs exhaled by blood samples collected from CRC affected subjects and blood samples collected from healthy ones (as controls), by using a novel sensor combination. The final goal is to find the sensors that perform the best discrimination between healthy and tumor affected subjects.

## 2. Results

**Figure 1:**
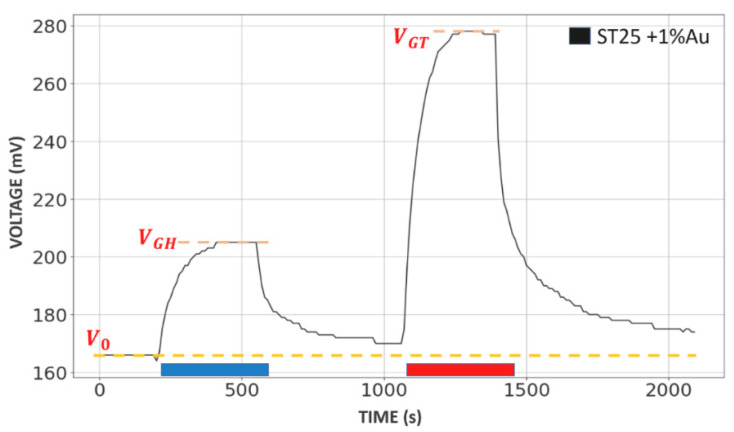
Example of an output voltage sensor signal. A ST25 + 1%Au sensor was exposed to a stream of clean and dry air (giving a steady voltage response of amplitude *V_0_*; orange dotted line) in between a stream of air polluted with the blood headspace volatile organic compounds (VOCs) of a healthy subject (HB; which timing is shown by the thick blue line; response attained a steady state amplitude *V_GH_*, orange dotted line) and of a tumor affected one (TB; thick red line; steady state amplitude *V_GT_*, orange dotted line). The steady state was considered to be achieved when voltage did not change more than 2/3 mV in 30 s.

A typical voltage sensor signal, generated by the electronics of SCENT B1 prototype and acquired by the dedicated software LSS4 (“Laboratorio Sensori—Sensori 4 v1.0”), is shown in Figure 1. The response was firstly characterized by a baseline of amplitude V_0_ when the sensor (ST25 + 1%Au in Figure 1) was exposed to a dry and clean air stream. A large voltage change occurred when it was exposed to the sample chamber headspace VOCs of a blood sample collected from a healthy subject (HB; the application timing is shown by the thick blue line, and is about 5 min), attaining a steady state amplitude *V_GH_*, and an even larger voltage change when it was exposed to a blood sample collected from a tumor affected one (TB; thick red line), attaining a steady state amplitude *V_GT_* (Figure 1).

**Figure 2:**
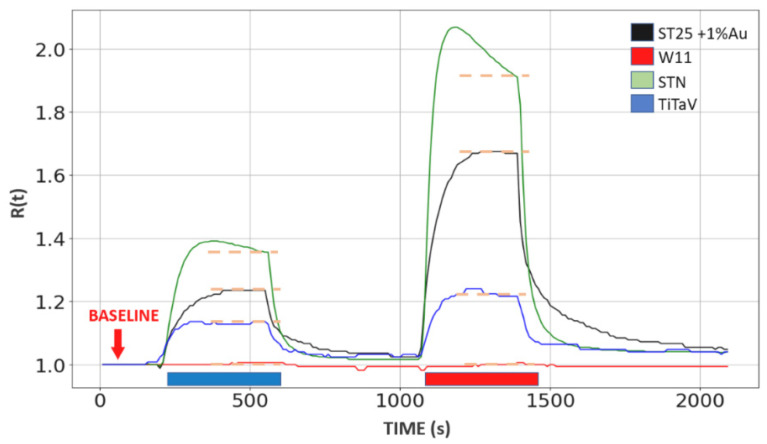
Computed response amplitude of the four sensors. The ST25, W11, STN and TiTaV sensor responses (represented by black, red, green and blue curves, respectively) to the HB exhalation (thick blue line) and of a TB one (thick red line); average steady state amplitudes are shown by the orange dotted lines.

Sensor response R(t) (Figure 2) was computed by dividing its voltage response *V_G_(t)* when it was exposed to a blood sample by the baseline *V_0_(t)*, exploiting the relation:

(1) $$R\left(t\right)=\frac{V_{G}\left(t\right)}{V_{0}\left(t\right)}$$

The time-dependence of the baseline indicate that it could slightly drift during the experiment.

**Figure 3:**
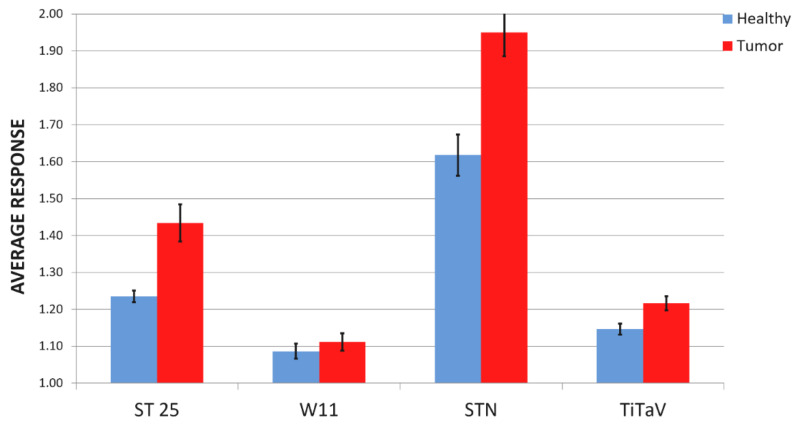
Basic statistical analysis of sensor responses. Average response and standard error of the response of the four sensors to HB (blue bars, *n* = 23) and to the exhalations of TB (red bars, *n* = 25).

The average *R(t)* amplitude plot at the steady state and its standard error were estimated for each sensor, when exposed to the TB headspace VOCs (*n* = 25; AVE. T, Table 1; Figure 3 red bars) and HB ones (*n* = 23; AVE. H, Table 1; Figure 3 blue bars). The “discriminating power” (i.e., how much the TB average responses are different than the HB ones in percentage; D.P., Table 1) of each sensor can be calculated from their average responses (Figure 3) by the Equation (2):

(2) $$D.P.=100\times \left(\frac{R_{T}}{R_{H}}-1\right)$$

where *R_T_* and *R_H_* are the average sensor responses to TB and HB respectively. The confidence intervals given by the Student’s t-test, were calculated for the TB and HB responses (C.I. T, C.I. H respectively, Table 1), with 90% of confidence level (α = 0.1).

**Figure 4:**
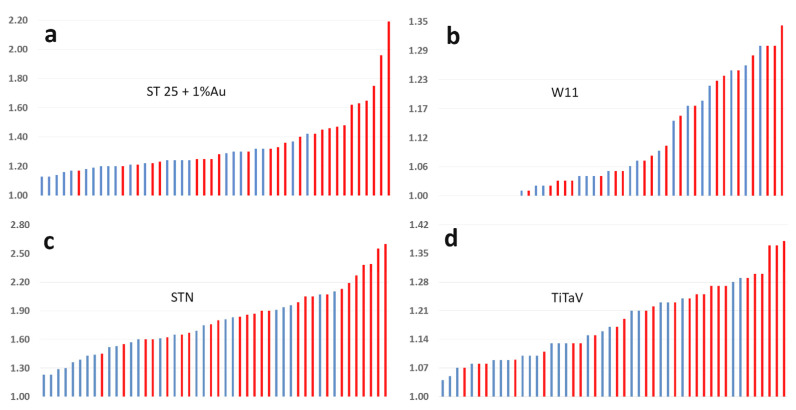
One sensor approach. Responses of the four sensors, sorted by their amplitude and plotted in ascending order. Blue and red bars are the sensor responses to healthy subject blood samples and tumor ones, respectively. (**a**), ST25 sensor; (**b**), W11; (**c**), STN; (**d**), TiTaV.

Through the one sensor approach (Figure 4), i.e., plotting all the responses (*n* = 48) per sensor in ascending order, it is easy to conclude at first sight that each sensor, excluded W11, gave the largest responses when exposed to TB headspace VOCs. 

In order to estimate the sensor “diagnostic” capability, i.e., the accuracy in discriminating TB and HB, sensitivity (i.e., the ability to recognize true positives; Equation (3a) and specificity (i.e., the ability to recognize true negatives; Equation (3b) parameters, computed through the relations Equations (3a) and (3b), were used to perform the ROC (receiver operating characteristic) analysis.

(3a) $$\mathrm{Sensitivity}=\frac{TP}{\left(TP+FN\right)}$$

(3b) $$\mathrm{Specificity}=\frac{TN}{\left(TN+FP\right)}$$

where *TP* and *TN* are the number of true positives and true negatives, respectively (i.e., the number of the actual sick and healthy subjects, correctly recognized by a screening device); *FP* and *FN* are the number of false positive and of false negative, respectively (i.e., the number of the healthy and sick subjects, but recognized as positives and negatives by a screening device respectively).

**Figure 5:**
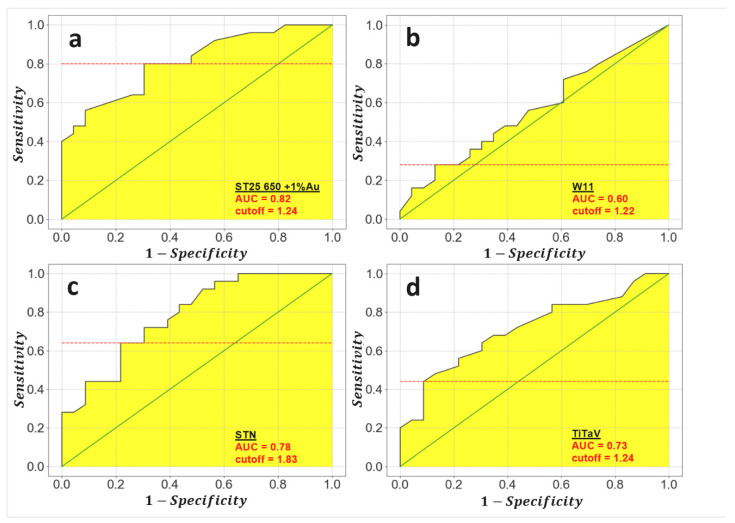
Evaluation of the threshold response amplitude discriminating between tumor affected and healthy affected subjects (TB and HB). Plots of the ROC (receiver operating characteristic) curves of the four sensors (black lines), the equity line (green), and the threshold amplitude computed by the maximum Youden’s index (red dashed lines). The AUC (area under curve) is highlighted in yellow. (**a**), ST25 sensor; (**b**), W11; (**c**), STN; (**d**), TiTaV.

The ROC analysis consists in the plot of sensitivity vs. 1−specificity; (Figure 5) the area under the resulting curve (AUC) ranges between 0.5 and 1.0, representing no and perfect diagnostic ability, respectively (i.e., closer is the AUC to one, higher is the test accuracy). The calculated AUC for each sensor employed here were (in decreasing order): 0.82 (ST25), 0.78 (STN), 0.73 (TiTaV), and 0.60 (W11; (Figure 5). The cut-off (or threshold) amplitude for each sensor that discriminates between HB and TB with the best sensitivity and specificity compromise can be calculated on the basis of the maximum Youden’s index. The latter is defined as the maximum vertical distance between the equity line (i.e., the points where sensitivity = 1−specificity) and the ROC curve [26].

## 3. Discussion

All sensors gave a larger voltage change in the presence of TB with respect to HB, although with diverse amplitudes. A qualitative analysis of the four sensor responses plotted in ascending order (Figure 4), indicates instead that ST25 + 1% Au is the best sensor to discriminate between TB and HB, followed by STN and TiTaV; in any case, both analyses indicate that all sensors, but W11, clearly discriminated between TB and HB. Furthermore, the confidence intervals C.I. T and C.I. H (Table 1), computed through the Student’s t-test, were clearly separated for ST25 and STN sensors, making them the best choice among the sensors used here. A more thoughtful scrutiny of the sensor responses, to assess their “diagnostic” accuracy, is provided by the ROC analysis (Figure 5). The larger is the AUC (Figure 5 and Table 2), the more accurate is the discrimination ability of a sensor: on this basis, ST25 and STN sensors exhibit the best compromise between sensitivity and specificity (Table 2, last two columns). From the ROC curves it is possible to calculate, on the basis of the maximum Youden’s index, the cut-off response amplitude for each sensor (Table 2), necessary to discriminate between HB and TB with the best compromise between specificity and sensitivity.

ST25 and STN sensors, both based on tin-oxide and titanium-oxide semiconducting nanoparticles, have demonstrated to be the best sensors (among the tested ones) to reveal blood samples headspace cancer VOCs. However, the less performing sensor in this study (W11) was instead very suitable in detecting VOCs exhaled from tumor biopsies surgically removed from colon [24]. In general, therefore, a sensor could be suitable or not to detect a particular tumor, depending upon the sample kind (i.e., blood, biopsy, saliva, breath, urine, etc.) or, for the same sample kind, to what tumor it is targeted (i.e., colon, brain, kidney, etc.).

Considering the unsuitability of W11 sensor, and the positive but poor results of TiTaV one to detect blood VOCs, a new prototype is under development where these two sensors will be replaced with more suitable ones. 

## 4. Materials and Methods 

### 4.1. Used Nanostructured MOX Sensors

The sensors used here are based on semiconductor materials able to detect gases with extremely low detection limit (up to tens of parts per billion), but still having a reliable and quick response. Their sensitive material is made of metal-oxide nanoparticles (MOX) capable of varying their conductivity if ionized gas particles are reversibly adsorbed on their surface [27,28,29]. 

**Figure 6:**
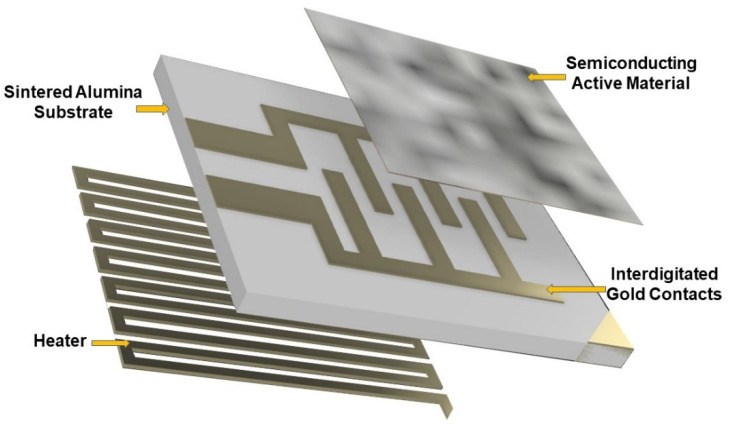
Three-dimensional (3D) representation of components constituting a metal oxide (MOX) sensor.

MOX sensors are typically made by three components (Figure 6):

- A substrate, a stiff and insulating layer (here made of sintered alumina), hosting two interdigitated gold contacts, necessary to connect the sensor to the transduction circuit;
- A sensitive material (or active material), a porous thick film (~20 µm of thickness) of semiconducting MOX nanoparticles (spherical nanograins with size ranging between 50 and 200 nm).
- A heater, a platinum coil necessary to activate the sensor at the proper working temperature which is controlled by means of the current flowing in the coil.

All the sensors used here are entirely designed, produced, and assembled in the “Laboratorio Sensori” of the University of Ferrara, Italy, using techniques commonly employed to produce thick film MOX sensors. The MOX nano powder, composing the sensing material, is synthesized through sol gel technique [30], then transformed in a printable viscous paste adding a small amount of organic vehicles and glass frit (a mixture of glassy silicon oxides). This final metal-oxide nanostructured paste is distributed on the alumina substrate between the two gold interdigitated contacts by means of a screen-printing machine (Aurel C920). The printed substrate is subjected to drying and firing thermal processes: the former takes place at low temperatures (about 100 °C) and the latter at higher ones (up to 850 °C). Finally, it is welded by a thermo-compression bonding technique on a four pin TO-39 socket, suitable to connect the sensor into the transduction circuit and to make it easily replaceable [31]. 

### 4.2. SCENT B1 Prototype Working Principle

**Figure 7:**
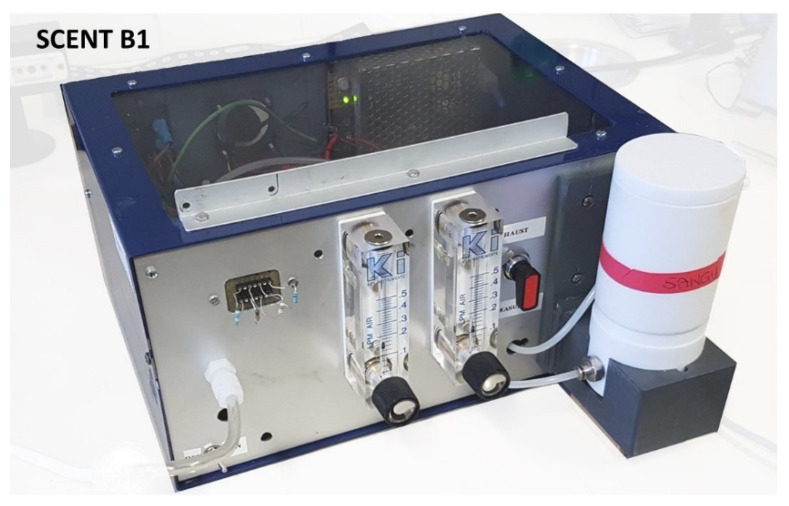
SCENT B1 device.

**Figure 8:**
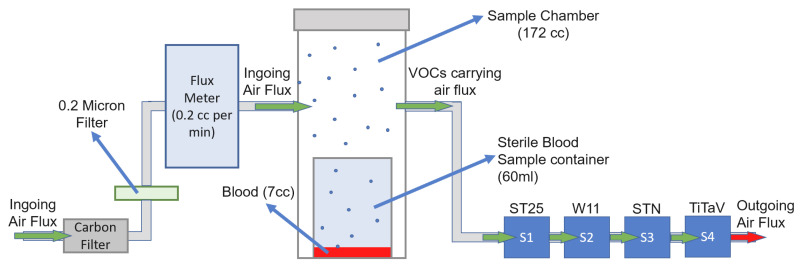
Block scheme of SCENT B1 pneumatic system.

SCENT B1 [21] (Figure 7) is made by a pneumatic air system and an electronic circuit. The pneumatic system (Figure 8) draws air from the environment and blows with a constant pressure into a carbon filter (to stabilize as much as possible the air humidity and temperature) and a 0.2-micron filter (to remove pollutants like particulate matter or aerosols) by means of an electronic pump.

This clean air flux can be guided directly to the sensors (whose response in this condition is considered the “baseline”, detailed below), or into the sample chamber (where it carries the sample headspace chemical compounds) before reaching the sensors by means of a three-way valve. The electronic circuit converts the resistance change of each sensor active film in a potential difference by means of an inverting operational amplifier. The acquired voltage vs. time is plotted by using a software developed ad hoc by SCENT S.r.l. research team, then transformed in a response R(t) vs. time plot exploiting the relation (1), to obtain a plot independent by the measured physical quantity and the baseline amplitude, generally diverse for each sensor [24].

### 4.3. SCENT B1 Hosted Sensors

The VOCs composing the headspace of TB and HB have been measured here by using the patented prototype SCENT B1 [21]. The MOX sensors equipping this prototype, carefully selected after many laboratory tests (carried out for many years on pure gases, feces, and human cancer biopsies at the “Laboratorio Sensori, LS” of University of Ferrara, Italy [see 14, 17-24]), are:

- ST 25 + 1%Au, based on tin and titanium (25%) oxides and enriched with gold nanoparticles (1%);
- W11, based on pure tungsten oxide;
- STN, based on the same percentage of tin, titanium, and niobium oxides;
- TiTaV, based on titanium, tantalum and vanadium oxides.

All the sensors used here worked at the temperature of 450 °C to fully activate the sensing layer and maximize the sensor detection capabilities [32].

### 4.4. Patient Recruitment and Sample Analysis

Blood samples were collected in K3 EDTA sample tubes of 7 cc of volume, from CRC affected and healthy subjects (used as controls) by the medical staff of the Hospital of Cona, Ferrara, Italy. The samples were kept at room temperature, transferred in a sterile container (60 mL), placed in the sample chamber (172 cc; Figure 8), and measured after 20/30 s to generate the headspace. Each blood sample was measured only once because testing different samples collected from the same patient gave the same results; moreover, we tried to minimize the amount of blood drawn from a patient. We did not measure the same blood sample many times to avoid possible sample alteration.

These subjects were selected with the following constraints:

- Age over eighteen years old;
- Both sexes;
- No any neoadjuvant therapy;
- No pregnant women.

Other factors, as sex, age, tumor stage and grade, were not correlated with the sensor responses because it would need a very large number of subjects to have statistical significance. CRC and healthy subjects (used as controls) were about 50% male and 50% female; the average age of CRC patients was higher than the one of the healthy subjects, because this pathology, as widely described in the Introduction, affects mainly people older than 50 years. Moreover, the young age of the healthy subjects ensures the minimization of the number of the risk factors. Nevertheless, considering the encouraging results obtained here, a new research study has been started employing a much larger number of subjects, aimed to follow the patients pre- and post-surgery, and the results will be correlated with the above-mentioned factors. 

The blood samples and the patient’s related clinical data were gathered upon written consent from patients, before undergoing to open or laparoscopic surgery as primary CRC treatment. The trial protocol and the informed consent form were presented, accepted, and retrospectively registered by the Ethical Committee of the District of Ferrara, with trial number 170484, on 13 July 2017.

The blood sample (collected about one hour before) was carefully poured into a stool specimen container avoiding any contamination; the container was then placed inside the sample chamber of SCENT B1 device to be measured at room temperature.

## 5. Conclusions

The work consists in a feasibility study to select the most suitable sensors and statistical approach to discriminate between TB and HB headspace VOCs. Each sensor here employed, but W11, proved to be capable of discriminating between HB and TB, with different accuracy. The sensors used here for the first time on blood samples, whose responses were analyzed with receiver operating characteristic method, led to identify the ST25 and STN as the best sensors (AUC: 0.82 and 0.78, respectively), whose sensitivity resulted of 80.0, 69.6 and specificity of 64.0, 78.3, respectively, exhibiting well separated confidence intervals. Considering the costs, easiness and non-invasiveness, our test is comparable with only fecal immunochemical test, although its sensitivity in CRC detection is not clearly known, because the blood presence in the stools is not always correlated to this pathology. These encouraging results led us to undertake a further study, replacing W11 sensor with a more suitable one, and involving many more cases and a follow up protocol aimed to monitor the patients after surgery, to reveal possible tumor relapses. The final goal consists in building a pre- and post-screening device that, combined to existing screening methods (such as FIT analysis and conventional colonoscopy in the case of CRC) could improve tumor detection efficacy and accuracy, also extending its application to other types of tumor. 

## 6. Patents

The Scent B1 device is patented in Italy with patent number: 102015000057717 [21].

## Figures and Tables

**Table 1:** Average sensor responses statistical analysis.

SENSOR	AVE. T	AVE. H	D.P. (%)	C.I. T	C.I. H
ST25	1.43 ± 0.05	1.24 ± 0.02	16.09 ± 4.79	1.35–1.52	1.21–1.26
W11	1.11 ± 0.02	1.09 ± 0.02	2.31 ± 3.98	1.07–1.15	1.05–1.12
STN	1.95 ± 0.06	1.62 ± 0.06	20.51 ± 6.69	1.84–2.06	1.52–1.72
TiTaV	1.22 ± 0.02	1.15 ± 0.02	6.13 ± 2.88	1.18–1.25	1.12–1.17

**Table 2:** Sensor performances on the basis of the ROC analysis.

SENSOR	AUC	CUT-OFF	SENS.%	SPEC.%
ST25	0.82	1.24	80.0	69.6
STN	0.78	1.83	64.0	78.3
TiTaV	0.73	1.24	44.0	91.3
W11	0.60	1.22	28.0	87.0

## Data Availability

The data presented in this study are available on request from the corresponding author. The data are not publicly available due to privacy and ethical restrictions (GDPR, (UE) n. 2016/679).

## Sample Availability

Samples of the compounds are available from the authors.
